# Distinct cerebellar regions for body motion discrimination

**DOI:** 10.1093/scan/nsz088

**Published:** 2019-12-10

**Authors:** Chiara Ferrari, Andrea Ciricugno, Lorella Battelli, Emily D Grossman, Zaira Cattaneo

**Affiliations:** Department of Brain and Behavioral Sciences, University of Pavia, Pavia 27100, Italy; IRCCS Mondino Foundation, Pavia 27100, Italy; Center for Neuroscience and Cognitive Systems, Istituto Italiano di Tecnologia, Rovereto 38068, Italy; Berenson-Allen Center for Noninvasive Brain Stimulation, Harvard Medical School, Boston 02155, MA, USA; Department of Cognitive Sciences, University of California, Irvine 92617, CA, USA

**Keywords:** biological motion perception, TMS, cerebellum, social cognition

## Abstract

Visual processing of human movements is critical for adaptive social behavior. Cerebellar activations have been observed during biological motion discrimination in prior neuroimaging studies, and cerebellar lesions may be detrimental for this task. However, whether the cerebellum plays a *causal* role in biological motion discrimination has never been tested. Here, we addressed this issue in three different experiments by interfering with the posterior cerebellar lobe using transcranial magnetic stimulation (TMS) during a biological discrimination task. In Experiments 1 and 2, we found that TMS delivered at onset of the visual stimuli over the vermis (vermal lobule VI), but not over the left cerebellar hemisphere (left lobule VI/Crus I), interfered with participants’ ability to distinguish biological from scrambled motion compared to stimulation of a control site (vertex). Interestingly, when stimulation was delivered at a later time point (300 ms after stimulus onset), participants performed worse when TMS was delivered over the left cerebellar hemisphere compared to the vermis and the vertex (Experiment 3). Our data show that the posterior cerebellum is causally involved in biological motion discrimination and suggest that different sectors of the posterior cerebellar lobe may contribute to the task at different time points.

## Introduction

A key component of daily social interactions is the ability to perceive other individuals’ body movements, in order to understand their actions but also their intentions and emotions. While we can identify actions, intentions and emotions of others even if this information is conveyed by only a handful of briefly presented points (typically used for biological motion experiments, Thornton *et al*., 2014; Lapenta *et al*., 2017), there are many pathological conditions in which this ability can be profoundly deteriorated (such as in autism spectrum disorder, Kaiser & Pelphrey, 2012; see also Urgesi *et al*., 2014). Cortical areas traditionally associated to biological motion processing are the ventral premotor cortex (Saygin *et al*., 2004; van Kemenade *et al*., 2012; Avenanti *et al*., 2013; Borgomaneri *et al*., 2015), the posterior parietal cortex (Battelli *et al*., 2003) and the posterior superior temporal sulcus (pSTS) (Grossman *et al*., 2000, 2005; Sokolov *et al*., 2018). In particular, thanks to its connection with both the ventral and dorsal pathway (Puce & Perrett, 2003), STS is supposed to integrate form and motion information (Allison *et al*., 2000; but see Vangeneugden *et al*., 2014) and to be involved in processing animacy, agency, intentions and emotions of others revealed by actions and movements (Carrington & Bailey, 2009). Interestingly, consistent neuroimaging and patients’ findings point to the cerebellum as a critical node of the cerebral network subtending biological motion processing (Grossman *et al*., 2000; Sokolov *et al*., 2010, 2012, 2014; Vaina *et al*., 2001; Jack & Pelphrey, 2015; Jack *et al*., 2017; for a review on the role of the cerebellum in perceptual processes, see Baumann *et al*., 2015). The functional significance of the cerebellar contribution to the perception of biological motion has been so far little investigated. In light of neuroimaging evidence reporting connections between the cerebellum, in particular the posterior cerebellar lobe, and STS (Jack *et al*., 2011, 2017; Sokolov *et al*., 2012, 2014, 2018; Jack & Pelphrey, 2015), this structure may be a critical node of an extended network mediating perception of biological motion, and possibly underlying other processes—such as emotion and action recognition—relevant for social understanding (Cattaneo *et al*., 2012; Van Overwalle *et al*., 2014; Ferrari *et al*., 2018a, 2019).

Available findings suggest that different cerebellar sectors are recruited during biological motion processing, both in the anterior and the posterior cerebellar lobes, although posterior activations have been more consistently reported (e.g. Vaina *et al*., 2001; Sokolov *et al*., 2012; Jack *et al*., 2017). Specifically, in an fMRI study investigating brain areas involved in biological motion processing, Grossman *et al.* (2000) showed a preferential response to biological compared to scrambled motion (during a one-back repetition task) in the anterior portion of the cerebellum, starting near the midline. However, that study did not specifically focus on the cerebellum and only the more superior part of the cerebellum was scanned. Vaina *et al.* (2001) found selective activations in the cerebellar left posterior quadrangular lobule (lobule VI) when participants had to discriminate a ‘walker’ from a ‘scrambled walker’ compared to motion direction discrimination or gender discrimination of biological stimuli (faces). Accordingly, an earlier PET study found evidence of a left posterior cerebellar activation during viewing of biological *vs*. random motion (Bonda *et al*., 1996). Other neuroimaging studies specifically focusing on the cerebellum reported consistent activations in the posterior cerebellar lobe during biological motion viewing or discrimination (Sokolov *et al*., 2012; Jack & Pelphrey, 2015; Jack *et al*., 2017). Specifically, Jack & Pelphrey (2015) found significant cerebellar activity associated with viewing animate *vs* random movement in different sectors of the posterior cerebellar lobe, including lobule VIIb, Crus I/II bilaterally, vermis IX–X and left lobule IX. Interestingly, activity in left Crus I (and adjacent left lobule VI) was positively associated with the degree to which participants described the stimuli in social-affective *vs* motion-related terms (Jack & Pelphrey, 2015). Jack *et al.* (2017) reported that typically developing individuals (compared to those with autism spectrum disorder) recruit regions throughout the cerebellar posterior lobe (especially bilateral sectors of lobule VI) while viewing biological motion. Finally, Sokolov and colleagues observed increased activity in response to human biological as compared to scrambled motion selectively in the left posterior cerebellar hemisphere, specifically in Crus I and lobule VIIB (Sokolov *et al*., 2012). The preferential activation in the left cerebellar hemisphere is likely to reflect the predominant role of the right (*vs*. left) STS in processing biological motion, with which the left posterior cerebellum (left lobule VI, left Crus I/II) has been found to be anatomically and functionally connected (e.g. Jack *et al*., 2011, 2017; Sokolov *et al*., 2012, 2014, 2018; Jack & Pelphrey, 2015).

The finding of a consistent involvement of the posterior cerebellar lobe in biological motion discrimination is interesting in light of the sensorimotor–cognitive dichotomy observed in cerebellar functions (for a recent review, see Schmahmann, 2019; see also Baumann *et al*., 2015). Indeed, whereas the anterior cerebellar lobe (lobules I–V, but also including adjacent parts of lobule VI and lobule VIII in the posterior cerebellar lobe) is mainly involved in mediating sensorimotor functions, topographically distinct regions within the posterior cerebellar lobe seem to mainly contribute to higher-level (cognitive) functions, including drawing social inferences and emotional processing (Schmahmann, 2019). Accordingly, whereas the anterior cerebellar lobe (but also adjacent regions of lobule VI) and lobule VIII are functionally connected with the sensorimotor cortices in the cerebrum, the cerebellar posterior lobe is reciprocally interconnected with cerebral association cortices and paralimbic areas (Buckner *et al*., 2011; Guell *et al*., 2018; for a review, see Stoodley & Schmahmann, 2018). In light of neuroimaging evidence reporting functional connections between the posterior cerebellar lobe and STS (Jack *et al*., 2011, 2017; Sokolov *et al*., 2012, 2014, 2018; Jack & Pelphrey, 2015), the posterior cerebellum may be a critical node of an extended network mediating processing of socially relevant information, including biological motion discrimination but also emotion attribution and action recognition (Cattaneo *et al*., 2012; Van Overwalle *et al*., 2014; Ferrari *et al*., 2018a, 2019).

The aim of the present study was to shed light by means of transcranial magnetic stimulation (TMS) on the specific *causal* contribution of the posterior cerebellum to biological motion perception. By interfering with ongoing neural activity, TMS allows to reveal the *causal* role of the targeted region in mediating a particular function/behavior, thus complementing the correlational nature of neuroimaging evidence. In three different experiments, participants were asked to discriminate biological from scrambled motion, while receiving TMS over the posterior cerebellum. We targeted both medial (vermal lobule VI) and lateral (left lobule VI/Crus I) sectors of the posterior cerebellar lobe based on prior neuroimaging evidence reporting both medial and lateral activations during biological motion discrimination (Grossman *et al*., 2000; Vaina *et al*., 2001; Sokolov *et al*., 2012, 2014; Jack *et al*., 2017). We expected TMS over the posterior cerebellum to interfere with biological motion discrimination compared to stimulation of a control site (vertex). Moreover, if lateral sectors of the posterior cerebellum are more involved than medial sectors in biological motion discrimination (e.g. Bonda *et al*., 1996; Sokolov *et al*., 2012, 2014; see also Sokolov *et al*., 2010 for patients’ evidence), TMS-induced interference should be more evident following stimulation of the lateral than medial posterior cerebellum.

### Experiment 1

In Experiment 1, participants performed a biological motion discrimination task on point-light animations while receiving (at stimulus onset) triple-pulse TMS on regions of the posterior cerebellum—vermal lobule VI and the left lateral cerebellum corresponding to lobule VI/Crus I—that were found to respond to biological motion discrimination in prior neuroimaging studies (e.g. Vaina *et al*., 2001; Sokolov *et al*., 2012; Jack *et al*., 2017).

## Methods

### Participants

Thirty-two Italian university students (7 M, mean age = 23.4 years, s.d. = 1.8) took part in Experiment 1. Before the experiment, each participant filled in a questionnaire (translated and adapted from Rossi *et al*., 2009) to evaluate compatibility with TMS. None of the volunteers had a history of neurological disorders or brain trauma or family history of epilepsy. Written informed consent was obtained from all participants before the experiment. The protocol was approved by the local ethics committee, and participants were treated in accordance with the Declaration of Helsinki.

**Figure 1:**
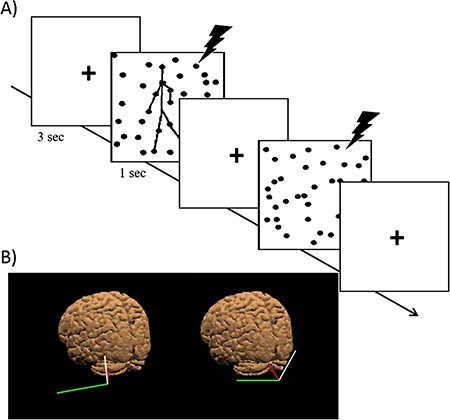
(**A**) The timeline of an experimental trial in Experiment 1. Each trial started with a fixation cross (3 s) followed by the animation (1 s) depicting either biological or scrambled motion. (**B**) Targeted sites on the scalp of one representative participant as shown by the neuronavigation system (SofTaxic 2.0, EMS, Bologna, Italy): (left side) posterior left cerebellar hemisphere (lobule VI/Crus I, *x* = −38, *y* = −66, *z* = −16, TAL); (right side) the vermis (Lobule VI, *x* = 0, *y* = −71, *z* = −14, TAL). The red line represents the magnetic field generated by the stimulator, and the white and green segments represent the longitudinal and lateral orientation of the coil, respectively.

### Stimuli and procedure

Participants were seated in front of a 19′ screen at an approximate distance of 57 cm. Figure 1A shows an example of the experimental trial. Stimuli consisted of point-light animations depicting either a biological figure in movement (performing various activities such as walking, kicking or throwing) or non-biological (‘scrambled’) motion created by randomizing the starting position of each dot of the biological-motion configuration within a region approximating the biological figure. Scrambled animations appear as a meaningless cloud of dots with some overall flow in common. Animations were identical to those used in Grossman *et al.* (2005). Light points were displayed as black dots against a white background (covering 3 × 6° of visual angle) and lasted for 1 s, spaced out by a fixation cross lasting for 3 s. For each animation, participants were instructed to discriminate biological from scrambled motion using a two-alternative forced choice response and responding with their right index and middle finger. Response keys were counterbalanced among participants. Participants were required to respond as fast as possible during the presentation of the animation. When participants could not respond within the end of the animation, a black screen appeared and it lasted until they responded. Each block consisted of 100 trials, 50 containing biological-motion animations and 50 containing scrambled-motion animations. Each block was repeated three times, once for each stimulation site (vertex, left cerebellum and vermis, see TMS section below for details), for a total of 300 trials. The order of the stimulation sites was counterbalanced across participants. Before the TMS experiment, we psychophysically measured participants’ threshold to biological motion discrimination as done in prior studies (Grossman *et al*., 2005). In the thresholding session, participants had to discriminate biological from scrambled motion (as in the real experiment). The number of noise dots was increased after two correct responses and decreased for each incorrect response (see Grossman *et al*., 2005 for details). Through this procedure, the optimal number of noise-dots leading to an accuracy of 70% was determined for each participant and kept fixed for the entire TMS experiment. The number of noise-dots tolerated averaged 73.4 dots (s.d. = 23.2). Matlab (MathWorks, Inc.) in conjunction with the Psychophysics Toolbox was used to display the animations and to record participants’ responses and response times (measured from the onset of the animation). The experiment took on average 1 h and 30 min (including instructions, fill-in of TMS questionnaire and informed consent, thresholding, neuronavigation and debriefing).

### TMS

Online neuronavigated TMS was performed with a Magstim Rapid2 stimulator (Magstim Co., Ltd, Whitland, UK) connected to a 70-mm butterfly coil. TMS was delivered over the left cerebellum, the vermis and the vertex (control site). At the beginning of the experimental session, single-pulse TMS was applied over the left M1 at increasing intensities to determine individual motor threshold (MT; see Hanajima *et al*., 2007, for methodological details on this standard procedure). MT was defined as the minimal intensity of the stimulator output that produces motor evoked potentials (MEPs, the motor response measured by applying electrodes to the hand muscles) with amplitude of at least 50 μV in the muscle with 50% probability (Petitet *et al*., 2015). Participants were stimulated at 100% of their MT. The intensity of stimulation was kept constant for the stimulation of all the three target sites and corresponded to an average of 50.0% of the maximum stimulator output (s.d. = 3.7) across the 32 participants. Triple-pulse 20 Hz TMS was delivered at the onset of the animation, covering the first 150 ms of the animation presentation, these parameters of stimulation effectively modulating behavioral responses in previous studies targeting the cerebellum (Koch *et al*., 2007; Gamond *et al*., 2017; Ferrari *et al*., 2018b).

The left cerebellum and the vermis were localized using stereotaxic navigation on individually estimated magnetic resonance images (MRIs) obtained through a 3D warping procedure fitting a high-resolution MRI template with the participant’s scalp model and craniometric points (SofTaxic 2.0, EMS, Bologna, Italy). This procedure has a global localization accuracy of about 5 mm, a level of precision close to that obtained using individual MRI scans (Carducci & Brusco, 2012), and it has been successfully used in many prior studies (e.g. Balconi & Ferrari, 2013; Cattaneo *et al*., 2014a; Ferrari *et al*., 2016, 2018a, 2018b, 2018c). Talairach coordinates (Talairach & Tournoux, 1988) for the targeted cerebellar loci were obtained from prior neuroimaging work and corresponded to peaks of activation during biological motion perception in the left posterior lobule VI/Crus I (TAL *x* = −38, *y* = −66, *z* = −16, Vaina *et al*., 2001; these TAL coordinates are very close to MNI coordinates for Crus I reported in Sokolov *et al*., 2014, *x* = −39, *y* = −56, *z* = −30), and vermal lobule VI (Tal *x* = 0, *y* = −71, *z* = −14, Baumann & Mattingley, 2010). The vertex was localized as the point falling half the distance between the nasion and the inion on the same midline. For cerebellar stimulation, the coil was placed tangentially to the scalp and held parallel to the midsagittal line with the handle pointing superiorly (see Figure 1B), in line with consistent evidence suggesting that this is an effective coil orientation to successfully modulate activity in cerebellar structures (Bijsterbosch *et al*., 2012). For the vertex stimulation, the coil was placed tangentially to the scalp and held parallel to the midsagittal line with the handle pointing backward. In order to minimize neck tension during the whole experiment, participants’ head was stabilized using a chinrest and few-minute breaks were allowed after each block. None of the participants reported phosphene perception, discomfort or adverse effects during TMS.

## Results

**Figure 2:**
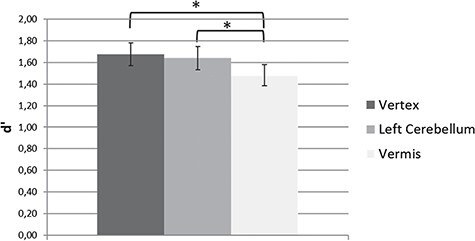
Mean *d*′ rates as a function of TMS site (vertex, left cerebellum and vermis) in Experiment 1. Triple-pulse 20 Hz TMS given at stimuli onset over the vermis affected participants’ ability to detect biological motion compared to stimulation of the left cerebellum and of the vertex (control condition). Error bars represent ±1 SEM. Asterisks denote a significant difference between TMS conditions.

The dependent variables were detection sensitivity values (*d*′, Macmillan & Creelman, 2004, see Figure 2) and mean correct reaction times (RTs).

A repeated-measures ANOVA carried out on mean *d*′ values with TMS site (left cerebellum, vermis and vertex) as a within-subject factor revealed a significant main effect of TMS, *F*(2,62) = 4.16, *P* = .020, η_p_^2^ = .12. Pairwise comparisons (Bonferroni–Holm correction applied) showed that TMS over the vermis significantly impaired participants’ ability to discriminate biological from scrambled motion compared to the vertex stimulation, *t*(31) = 2.71, *P* = .033 (Cohen’s *d* = .48) and compared to TMS over the left cerebellum, *t*(31) = 2.51, *P* = .034 (Cohen’s *d* = .44). No difference in performance was observed for TMS applied over the left cerebellum and the vertex, *t*(31) = .44, *P* = .66.

Mean correct RTs were 1148 ms (s.d. = 266) for vertex TMS, 1140 ms (s.d. = 272) for left cerebellum TMS and 1122 ms (s.d. = 257) for vermis TMS. A repeated-measures ANOVA carried out on mean correct RTs with TMS site (left cerebellum, vermis and vertex) as a within-subject factor revealed no significant main effect of TMS, *F*(2,62) = .89, *P* = .42.

### Experiment 2

Experiment 1 showed that interfering with activity in the vermis (but not in the left cerebellar sector) affected participants’ ability to discriminate biological from scrambled motion, suggesting that the vermis is *causally* involved in processing biological motion. In Experiment 2, we repeated the same task on a new group of participants using single-pulse TMS. Single-pulse TMS allows to reduce the possibility that the effects reported in Experiment 1 derived from indirect stimulation of primary visual cortex by repetitive TMS over the vermis (Renzi *et al*., 2014). Indeed, triple-pulse but not single-pulse TMS over the vermis has been found to elicit phosphene perception, suggesting spread of stimulation to nearby visual cortex (Renzi *et al*., 2014). Moreover, point-light animations were presented both in the upright standard orientation (as in Experiment 1) and upside-down. Display inversion is considered as a proper control condition in biological motion tasks because the body kinematics are preserved, and as such it has been used in previous fMRI (Grossman & Blake, 2001; Pavlova *et al*., 2017) and TMS studies (Grossman *et al*., 2005). While inverted biological motion can still be successfully discriminated from scrambled motion, discrimination can only be achieved with less masking noise as compared to upright animations (Grossman *et al*., 2005). In fact, upside-down moving bodies violate gravity forces and are therefore perceived as somehow unnatural (Sumi, 1984; Pavlova & Sokolov, 2000). Accordingly, inverted biological point-light animations drive significantly less activation in pSTS compared to upright animations (Grossman & Blake, 2001; see also Pavlova *et al*., 2005, 2017). In light of this, we hypothesized that TMS over the vermis should interfere less with discrimination of biological from scrambled motion in upside-down animations compared to upright (standard) ones.

## Methods

### Participants

Forty-eight Italian university students (16 M, mean age = 23.5 years, s.d. = 2.5) took part in Experiment 2. All participants were right-handed and had normal or corrected-to-normal vision. None of them had participated in Experiment 1. Inclusion criteria were the same as for Experiment 1. The protocol was approved by the local ethics committee, and participants were treated in accordance with the Declaration of Helsinki.

### Stimuli and procedure

Stimuli and procedure were similar to Experiment 1, with the exception that animations could be presented either in the upright canonical orientation as in Experiment 1 or in inverted orientation, obtained by flipping upright-animations around the *x*-axis (see Grossman *et al*., 2005 for details on this manipulation). Upright and inverted biological-motion animations were presented in separate blocks. Each block consisted of 60 trials, 30 containing biological-motion animations (either upright or inverted, depending on the condition) and 30 containing scrambled-motion animations. The upright and inverted blocks were repeated three times, once for each stimulation site (vertex, left cerebellum and vermis, see TMS section below for details) for a total of 360 trials. At the beginning of each block, participants were informed about the stimuli orientation; half of the participants started with the upright condition, and the other half with the inverted condition. The order of presentation of the upright and inverted blocks and of the stimulation sites was counterbalanced across participants. Prior to the TMS experiment, participants were thresholded (following the same procedure described in Experiment 1) separately for the upright and inverted conditions. In the upright condition, participants tolerated more than the triple of noise level (number of noise-dots tolerated: 73.6, s.d. = 23.4) than in the inverted condition (20.2 dots, s.d. = 10.3), in line with prior studies (Grossman *et al*., 2005). The experiment took on average 1 h and 45 min (including instructions, fill-in of TMS questionnaire and informed consent, thresholding, neuronavigation and debriefing).

### TMS

Online neuronavigated TMS was performed similarly to Experiment 1, but single-pulse TMS was used. In order to control for the effects of possible TMS-induced eye blinking (Corthout *et al*., 2011), in half of the participants TMS was given at the onset of the animation, in the other half 100 ms after the onset of the animation (participants were randomly assigned to one of the two timing conditions). Target sites were the same as Experiment 1. Intensity of stimulation was set at 100% of the MT defined with the same procedure of Experiment 1 and corresponded to a mean stimulation intensity of 50.8% of the maximum stimulator output (s.d. = 3.5). None of the participants reported phosphene perception, discomfort or adverse effects during TMS.

## Results

**Figure 3:**
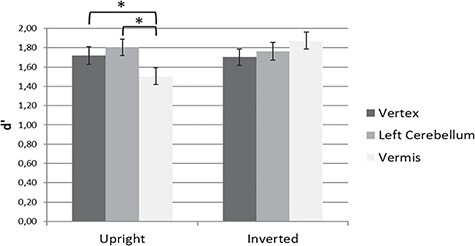
Mean *d*′ rates as a function of TMS site (vertex, left cerebellum and vermis) and Orientation (upright *vs*. inverted) in Experiment 2 (collapsed across timing of single-pulse TMS: at stimulus onset *vs*. 100 ms from onset). In the upright orientation, single-pulse TMS over the vermis affected participant’s ability to discriminate biological from scrambled motion compared to TMS over the left cerebellum and over the vertex. TMS had no effect on discrimination of biological motion when animations were presented upside-down. Error bars represent ±1 SEM. Asterisks denote a significant difference between TMS conditions.

Figure 3 shows participants’ mean *d*′ values. A repeated-measures ANOVA with TMS site (vertex, left cerebellum and vermis) and orientation (upright *vs*. inverted) as within-subject factors and TMS timing (onset *vs*. 100 ms) as a between-subject factor on mean *d*′ values revealed a significant interaction TMS by Orientation, *F*(2,92) = 9.40, *P* < .001, η_p_^2^ = .17. The analysis of the main effect of TMS site within each orientation revealed that TMS significantly affected discrimination of upright, *F*(2,94) = 7.50, *P* = .001, η_p_^2^ = .14 but not inverted animations, *F*(2,94) = 2.0, *P* = .14. Pairwise comparisons (Bonferroni–Holm correction applied) showed that in the upright orientation condition, TMS over the vermis lowered participants’ performance compared to TMS over the vertex, *t*(47) = 2.67, *P* = .020 (Cohen’s *d* = .39), and compared to TMS over the left cerebellum, *t*(47) = 3.83, *P* < .001 (Cohen’s *d* = .55). No difference in the discrimination of upright animations was observed between left cerebellum and vertex stimulation, *t*(47) = 1.0, *P* = .30. None of the other interactions or main effects reached significance (all *P* > .12).

Mean correct RTs for upright orientation were 1199 ms (s.d. = 258) for vertex, 1189 ms (s.d. = 307) for left cerebellum and 1207 ms (s.d. = 258) for vermis; for the inverted orientation, mean RTs were 1181 ms (s.d. = 321) for vertex, 1137 ms (s.d. = 286) for left cerebellum and 1144 ms (s.d. = 298) for vermis. The ANOVA performed on response times revealed a significant main effect of Orientation, *F*(1,46) = 6.54, *P* = .014, η_p_^2^ = .12, with participants being slightly faster in discriminating biological motion in inverted than upright animations. No other main effects or interaction effects reached significance (all *P* > .22).

### Experiment 3

Experiment 2 replicated the pattern of results of Experiment 1 with upright animations, ensuring that effects of vermis TMS in Experiment 1 did not depend on indirect stimulation of the visual cortex. Moreover, Experiment 2 showed that TMS over the vermis selectively affected discrimination of upright (*vs*. inverted) biological motion, suggesting that the vermis may house neurons selectively dedicated to code genuine human movements. Critically, in both experiments, we observed no significant behavioral effect of TMS when delivered over the left cerebellum. This finding may appear at odds with prior neuroimaging evidence reporting more consistent involvement of the left lateral compared to medial sectors of the posterior cerebellum during biological motion perception (Bonda *et al*., 1996; Sokolov *et al*., 2012, 2014; see also Sokolov *et al*., 2010 for patients’ evidence). Moreover, the left cerebellum is anatomically and functionally connected to the right STS (Jack *et al*., 2011, 2017; Sokolov *et al*., 2012, 2014, 2018; Jack & Pelphrey, 2015), a critical cortical area for biological motion processing. One possibility is that the lateral sectors of the cerebellum may intervene at later stages of biological motion processing, following STS response to biological motion animations that prior MEG and ERP studies estimated to occur between 170 and 350 ms after the animation onset (Hirai *et al*., 2003, 2005; Pavlova *et al*., 2004; Jokisch *et al*., 2005; Krakowski *et al*., 2011). To test for this hypothesis, in Experiment 3, we repeated the same paradigm used in Experiment 1 delivering TMS at a later time-point, specifically 300 ms after animation onset.

## Methods

### Participants

Thirty-two participants (9 M, mean age = 23.5 years, s.d. = 3.0) took part in Experiment 3. All participants were right-handed and had normal or corrected-to-normal vision. None of them had participated in either Experiment 1 or 2. Inclusion criteria were the same as for the previous experiments. The protocol was approved by the local ethics committee, and participants were treated in accordance with the Declaration of Helsinki.

### Procedure and TMS

Stimuli and procedure were identical to Experiment 1. Participants were required to discriminate biological *vs*. scrambled motion in point-light animations presented only in the canonical (upright) orientation. Before the TMS experiment, participants underwent a thresholding procedure, as in Experiment 1. The number of noise-dots tolerated during the experiment averaged 68.5 dots (s.d. = 32.4). TMS parameters were similar to Experiment 1, but this time triple-pulse 20 Hz TMS was delivered 300 ms after the onset of each animation, covering a time window ranging from 300 to 450 ms from the stimulus onset. The stimulation intensity (set at 100% of the individual MT) was 48.0% of the maximum stimulator output (s.d. = 3.6). None of the participants reported phosphene perception, discomfort or adverse effects during TMS. The experiment took on average 1 h and 30 min (including instructions, fill-in of TMS questionnaire and informed consent, thresholding, neuronavigation and debriefing).

## Results

**Figure 4:**
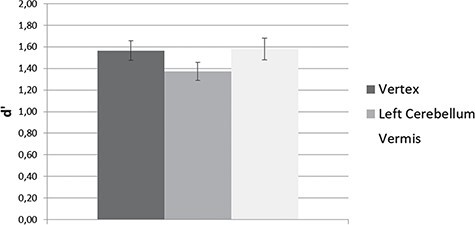
Mean *d*′ rates and as a function of TMS site (vertex, left cerebellum, and vermis) in Experiment 3. Triple-pulse 20 Hz TMS delivered 300 ms after the onset of each animation impaired discrimination of biological motion when delivered over the left cerebellum compared to the vermis (*P* = .056, Bonferroni–Holm correction applied) and the vertex (*P* = .057, Bonferroni–Holm correction applied), although the effect did not reach full significance. Error bars represent ±1 SEM.

Mean *d*′ values are shown in Figure 4. A repeated-measures ANOVA with TMS site (vertex, left cerebellum and vermis) as within-subjects factor on *d*′ values revealed a significant main effect of TMS, *F*(2,62) = 3.57, *P* = .034, η_p_^2^ = .10**.** Pairwise comparisons (Bonferroni–Holm correction applied) showed that TMS over the left cerebellum impaired participants’ ability to discriminate biological from scrambled motion compared to vertex stimulation, *t*(31) = 2.48, *P* = .057 (*P* = .019 not corrected) (Cohen’s *d* = .44) and compared to vermis stimulation, *t*(31) = 2.31, *P* = .056 (*P* = .028 not corrected) (Cohen’s *d* = .41). No difference in performance was observed between TMS over the vermis and the vertex, *t*(31) = .16, *P* = .87.

Mean correct RTs were 1256 ms (s.d. = 267) for vertex TMS, 1232 ms (s.d. = 245) for left cerebellum TMS and 1252 ms (s.d. = 299) for vermis TMS. The ANOVA carried out on RTs revealed no significant main effect of TMS site, *F*(2,62) = .54, *P* = .59.

## Discussion

In the present study, we investigated the contribution of vermal and (left) lateral sectors of the posterior cerebellar lobe in discriminating biological motion. In Experiments 1 and 2, TMS delivered at onset of the visual stimuli (or 100 ms after onset of the visual stimuli, Experiment 2) over vermal lobule VI, but not over the left cerebellar hemisphere (lobule VI/Crus I), interfered with biological motion discrimination. In turn, when stimulation was delivered 300 ms after stimulus onset (Experiment 3), TMS over the left hemisphere but not over the vermis tended to decrease participants’ ability to discriminate biological from scrambled motion. Overall, our data provide evidence for a causal involvement of the posterior cerebellum in biological motion discrimination, critically adding to prior correlational neuroimaging evidence (Grossman *et al*., 2000; Vaina *et al*., 2001; Sokolov *et al*., 2012; Jack & Pelphrey, 2015; Jack *et al*., 2017).

Lobule VI in the posterior cerebellum has been implicated in different tasks, being considered both part of the ‘sensorimotor’ and of the ‘cognitive’ cerebellum (see Schmahmann, 2019). TMS in Experiments 1 and 2 may have interfered with sensory-motor functions of vermal lobule VI, whereas lateral sectors of the posterior cerebellum may be more involved during high-order/cognitive tasks (see Klein *et al*., 2016, for a discussion on a possible medial-to-lateral gradient in cerebellar functions). The functional significance of the cerebellar contribution to sensory processing is still debated (for a critical discussion, see Baumann *et al*., 2015). One hypothesis is that the cerebellum controls the acquisition of sensory data, thus indirectly facilitating the computational efficiency of the rest of the brain (Bower, 1997; see Baumann *et al*., 2015 for a commentary), with TMS in our study possibly interfering with cerebellar monitoring of incoming visual information. This explanation would also account for disrupting effects of vermal TMS on other visual discrimination tasks, such as discrimination of (non-biological) motion direction (Cattaneo *et al*., 2014b). Another hypothesis is that the cerebellum aids visual information processing by making predictions in the form of internal models of sensory events (Cerminara *et al*., 2009; see Baumann *et al*., 2015; Sokolov *et al*., 2017, for discussion). Interfering by means of TMS with activity in medial sectors of lobule VI might have affected the implementation of these internal models (prediction of sensory outcomes) important for recognition of biological motion.

The hypothesis that TMS over vermal lobule VI may have specifically interfered with implementation of internal models related to prediction of sensory outcomes of biological motion is supported by the selective TMS effect we reported in Experiment 2 for animations appearing in the upright orientation. Indeed, data from Experiment 2 show that the medial posterior cerebellum (vermal lobule VI) is sensitive to the ‘authenticity’ of perceived biological motion. Previous neuroimaging studies have shown that biological motion-sensitive regions in the cerebrum—like STS—are recruited to a lesser extent when animations are presented upside-down (Grossman & Blake, 2001; Pavlova *et al*., 2017), possibly reflecting non-automatic access to the biological content of the displays. In fact, by contradicting gravity force, inverted animations are perceived as less natural, and they are thus harder to recognize than upright animations (Pavlova, 2011). Experiment 2 shows that medial sectors of the posterior cerebellum (similarly to STS) are sensitive to the authenticity of perceived biological motion, possibly generating perceptual internal models that rely on participants’ real-world experience. However, it is worth noting that the recruitment of the cerebellum may be related to the perceptual demands of a task (Brower *et al*., 1997; see also Baumann *et al*., 2015). In our task, the level of noise was higher in upright than in inverted animations (that were in fact discriminated faster) to ensure a similar level of accuracy across the two orientations (as in Grossman *et al*., 2005). Cerebellar TMS may have thus selectively affected discrimination of upright biological motion because here the level of noise was higher, a hypothesis that needs to be properly assessed by future research.

In Experiment 3, TMS delivered 300 ms from stimulus onset affected biological motion discrimination when the lateral (but not the medial) posterior cerebellum was targeted. What is the functional significance of the left cerebellar hemisphere recruitment at this stage of stimulus processing? Neuroimaging evidence has shown that the left cerebellum (in particular, left lobule VI, Crus I/II, VIIIb) is anatomically and functionally connected to the right posterior STS (e.g. Jack *et al*., 2011, 2017; Sokolov *et al*., 2012, 2014, 2018; Jack & Pelphrey, 2015), the core brain region deputed to the processing of biological motion (Allison *et al*., 2000; Grossman *et al*., 2000, 2005). ERP and MEG findings suggest that STS activates 170–350 ms after the onset of human point-light animations (as those used in the present study, Hirai *et al*., 2003; Krakowski *et al*., 2011; Pavlova *et al*., 2004). In light of this, the decrease in performance (albeit not reaching full significance when correcting for multiple-comparisons) induced by lateral cerebellum TMS delivered from 300 to 450 ms following stimulus onset may indicate interference with processing of feedback inputs from STS. It is worth noting here that feedback connections from associative cortices and the cerebellum might also account for a more general role of cerebellar hemispheres (specifically of lobule VI, Crus I/II, lobule VIIB) in social inferences (Guell *et al*., 2018; Ferrari *et al*., 2018a, 2019; Van Overwalle *et al*., 2019). Accordingly, abnormalities in the pathways connecting the lateral cerebellum to the cerebrum are associated to impairments in social abilities in neuropsychiatric diseases, like autistic spectrum disorders (Catani *et al*., 2008).

Some limitations need to be acknowledged when interpreting results of our study. One might argue that the impairment in biological motion discrimination following TMS over the vermis was due to stimulation spreading towards the nearby primary visual cortex that is involved in motion detection. Indeed, modeling studies have shown that effective stimulation of the cerebellum with a figure-of-eight coil (as the one we used) might be hampered by the anatomical features of the cerebellum and its distance from the skull, with a possible spread of cerebellar stimulation to the occipital regions (Bijsterbosch *et al*., 2012; Janssen *et al*., 2014; Van Dun *et al*., 2017, for a critical discussion). Also, our results may be due to TMS interference over eye movements (that were not recorded in our study). TMS over the cerebellum may indeed affect eye movements control (for a review, see Colnaghi *et al*., 2010), an effect that may be specifically relevant for stimulation of the vermis (Nitschke *et al*., 2004; Schraa-Tam *et al*., 2009; Glickstein *et al*., 2011). However, the selective effects of TMS in Experiment 2 for upright (*vs*. inverted) animations rules out the interpretation of our results in terms of unspecific effects of cerebellar TMS over visual processing. A further limitation to be considered is that the effect of TMS over the lateral cerebellum in Experiment 3 did not reach full significance; further testing is thus needed to clarify the specific contribution of lateral sectors of the posterior cerebellum to biological motion processing. Finally, one may wonder why in our study cerebellar TMS affected detection sensitivity but not response latencies. Since participants’ response times were overall long (exceeding 1 s), it is likely that error rate was more prone than response latency to be modulated by TMS, as also suggested by prior studies using a similar task (Grossman *et al*., 2005) and reporting selective effects of TMS on *d*′ measures.

In conclusion, our study offers some preliminary evidence that both medial and lateral sectors of the posterior cerebellar lobe contribute to perception of human bodies in motion, although at different stages of perceptual processing. Further research (also employing fMRI-guided TMS and a strict chronometric approach) is needed to clarify the specific topography and time course of the involvement of the posterior cerebellum to biological motion discrimination. Importantly, clarifying by means of TMS how the cerebellum contributes to the understanding of others’ movements and actions might have clinical implications for the understanding and treatment (Oberman & Enticott, 2015) of neuropsychiatric syndromes, such as autism spectrum disorder, in which impaired social abilities have been associated to anatomical and functional abnormalities in the cerebellum (Igelstrom *et al.*, 2017).

## Funding

This work has been supported by a Bando Ricerca Finalizzata (GR-2016-02363640) by the Italian Ministry of Health to Z.C.

*Conflict of interest*. None declared.
